# “Crossroads in Sepsis Research” Review Series Overview of the pathophysiology of sepsis

**DOI:** 10.1111/j.1582-4934.2008.00366.x

**Published:** 2008-05-09

**Authors:** Florea Lupu

**Affiliations:** Cardiovascular Biology Research Program, Oklahoma Medical Research FoundationOklahoma City, OK, USA


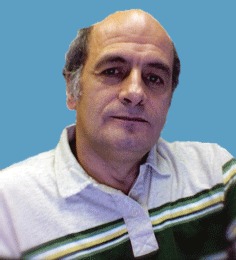


Sepsis is a life-threatening clinical syndrome that arises through the innate response to infection [1], and can appear as a complication of trauma, cancer or major elective surgery [2]. Despite great strides being made in understanding the pathophysiology and designing treatment for this disease, mortality rates still remain unacceptably high. Currently, sepsis rivals myocardial infarction as a common and potentially reversible cause of mortality in the developed world. At least 8,000,000 people develop this syndrome in the United States alone, and one-quarter of these die within 28 days of its onset. It is, therefore, of great importance to advance our understanding and hopefully find appropriate therapy for this major, and increasingly frequent medical problem.

**Fig. 1:**
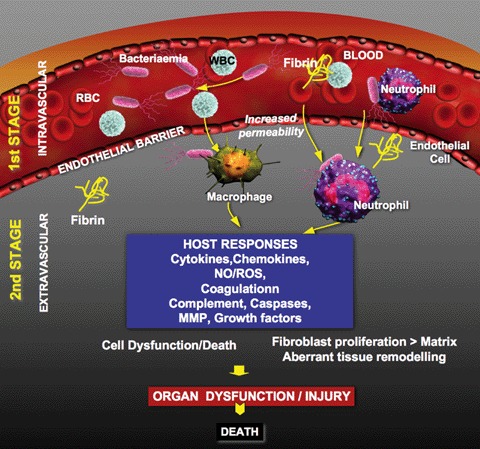
Diagram summarizing the stages and tissue-specific events controlling the progression of sepsis.

The pathogenesis of sepsis and its accompanying systemic inflammatory response syndrome (SIRS) reflect the inability of the body to regulate the immune response [3] (Fig. 1). Sepsis initiates as microbial components are recognized by soluble or cell-bound pattern recognition molecules or receptors, such as CD14 and Toll-like receptors (TLRs). Activation of these receptors induces the transcription of inflammatory and immune response genes, often *via* nuclear factor-kB (NF-kB), and establishes a hyperactive inflammatory response. Endothelial and epithelial cells, as well as neutrophils, macrophages and lymphocytes, produce the powerful pro-inflammatory mediators, such as TNF-α, interleukin (IL)-6, IL-1 and IL-8 [4]. Neutrophils and macrophages respond to these mediators by releasing granular enzymes and producing reactive oxygen species (ROS) such as H_2_O_2_, which, although effective in killing bacteria, also damages tissues and may produce increased vascular permeability and organ injury.

Besides inflammation, sepsis triggers other homeostatic systems, including those controlling blood coagulation and complement activation. During sepsis, both the coagulation system and the cells that regulate it are strongly activated, leading to disseminated intravascular coagulation (DIC), intravascular fibrin deposition and microthrombi, severe depletion of platelets and coagulation factors, and diffuse widespread bleeding [5]. Changes in vascular tonus and endothelial permeability lead to extravascular plasma escape (capillary leak), which enhances the viscosity and decreases the flow rate and hydrostatic pressure of the blood. All these can lead to tissue hypo-perfusion, hypoxia and ischaemia that further develop into organ failure and death [6, 7]. The recognition of the links between the coagulation system and the immune response [5, 8] advanced the understanding sepsis pathophysiology and led to the development of the only specific anti-sepsis treatment currently available, recombinant human activated protein C (APC) produced and marketed by Eli Lilly (Indianapolis, IN, USA) as Xigris®.

While early restoration of blood flow to ischaemic tissue is essential to halt the progression of cellular injury associated with decreased oxygen and nutrient delivery, late reperfusion of ischaemic tissue initiates a series of detrimental reactions, broadly named ischaemia-reperfusion (IR) injury [9]. This is a result of intense inflammation and oxidative damage induced by ROS, nitrogen metabolites and inflammatory leucocytes [10]. Reperfusion in a variety of organs triggers production of acute-phase proteins (*e.g.* CRP), a second round of pro-inflammatory mediators and activates defence mechanisms such as the complement system, leading to widespread deposition of complement complexes in the microcirculation [11]. While during early stages of sepsis complement is an important defence mechanism, helping to clear invading bacteria; however, complement activation ultimately contributes to and amplifies the IR injury during late stages [12]. Complement split products (C3a and C5a) enhance cytokine and chemokine production, elicit leucocyte chemotaxis and activation, promote ROS generation and adhesion molecule expression [13].

Currently, sepsis therapy is mainly limited to measures directed at its infectious causes (*e.g.* antibiotics, surgical and supportive therapies) rather than modifying the pathophysiologic processes responsible for its initiation and progression [14]. So far, most of single-hit therapeutic strategies have failed. Despite appearing as ideal targets, inhibition of early inflammatory mediators (TNF-apha and IL-1) showed no efficacy in clinical trials [15]. Since sepsis is a multi-stage and multi-factorial disease, complex interdisciplinary investigations leading to multi-hit therapeutic strategies must be developed.

Therefore, it is timely to have a multifaceted conversation on the pathophysiology of sepsis. *The Journal of Cellular and Molecular Medicine*, a widely respected forum for translational medical research had initiated this new series of state-of-the-art reviews focused on interdisciplinary aspects of the pathophysiology of sepsis. Recognized scientists will critically analyse the current status of sepsis research, with a particular focus on its interface with inflammation, innate immunity, coagulation and complement activation, as well as on the transitional aspects of animal model research and novel therapeutic approaches.
